# A study on predicting impaired fasting glucose risk in Chinese adults based on individual characteristics

**DOI:** 10.3389/fmed.2025.1584626

**Published:** 2025-06-09

**Authors:** Yijun Lin, Wenxu Wu, Xiaoyan Liang, Liping Zhou, Gan Li, Cuiling Kang, Wuzhen Li, Chunyi Huang, Feng Tian

**Affiliations:** ^1^Department of Health Management Centre, The Eighth Affiliated Hospital of Southern Medical University (The First People's Hospital of Shunde), Foshan, Guangdong, China; ^2^Department of Health Examination Center, The First People's Hospital of Nanning, Nanning, China; ^3^Department of Health Examination Center, The Fifth Affiliated Hospital of Guangxi Medical University, Nanning, China; ^4^School of Politics and Public Administration, South China Normal University, Guangzhou, Guangdong, China

**Keywords:** impaired fasting glucose, nomogram, risk score, prediction performance, LASSO

## Abstract

**Introduction:**

This study aimed to develop a nomogram for early detection of impaired fasting glucose (IFG), predicting the 5-year risk in Chinese adults due to its link to various diseases.

**Materials and methods:**

This retrospective cohort study included 28,875 participants without IFG at baseline, randomly divided them to a training set and a validation set. We developed four predictive models—LASSO, full, stepwise, and MFP—ultimately selecting the LASSO model for nomogram development due to its simplicity and predictive performance. Four prediction model performance was assessed through ROC analysis, calibration curves, and decision curve analysis, with external validation using Shunde Hospital (*n* = 18,618) and NHANES (*n* = 2,038) dataset.

**Results:**

We developed a nomogram to predict the risk of IFG by incorporating parameters including age, body mass index (BMI), systolic blood pressure (SBP), fasting plasma glucose (FPG), and triglycerides (TG), which demonstrated performance with AUCs of 0.8167 and 0.8155 in the training and validation set, respectively. External validation achieved AUC 0.9665 (Shunde Hospital dataset) and 0.9171 (NHANES).

**Conclusions:**

Our nomogram provides a personalized, validated approach for assessing 5-year IFG risk in Chinese adults, offering a practical screening tool for primary healthcare and resource-constrained environments.

## Introduction

Diabetes has become one of the most serious public health challenges in the world, and its incidence and prevalence continue to rise globally (1). In 2024, ~589 million adults aged 20–79 worldwide are suffering from diabetes globally, representing 11.1% of this age group; this figure is expected to rise to 853 million by 2050, accounting for 13.0% (2). According to data from the 11th edition of the International Diabetes Alliance (IDF) Diabetes Atlas, although the prevalence of diabetes in China is very high, there are indeed a large number of undiagnosed cases, and the undiagnosed rate of diabetes is as high as 49.7% (2). The American Diabetes Association (ADA) acknowledged impaired fasting glucose (IFG) as an indicator of prediabetes as early as 1997 (3). The incidence of IFG globally was 5.8% in 2021, accounting for 298 million individuals. It is anticipated that this figure will increase to 6.5%, representing 414 million individuals, by the year 2045 (4). Often without noticeable symptoms, IFG serves as a sign of a hidden and potentially dangerous early stage of abnormal glucose metabolism (5). Consequently, IFG may be easily disregarded, yet it is intricately associated with type 2 diabetes mellitus (4) and cardiovascular diseases (CVD) (6), as well as cardiometabolic multimorbidity (7), heart failure (8), chronic kidney disease (9), and cerebral hemorrhage (10).

In recent years, many risk prediction models for diabetes have been developed (11–13). The Finnish Diabetes Risk Score (14) (FINDRISC) and the ADA risk assessment tools (15) are two widely used diabetes risk prediction models. While FINDRISC is mainly designed for European populations without verified applicability to the Chinese population, the ADA risk tool emphasizes diabetes risk but does not offer a specific approach for predicting IFG. Few studies have specifically focused on modeling the risk of IFG, and most existing research primarily explores its risk factors through cross-sectional analysis (16). Therefore, it is crucial to develop reliable risk assessment models that enable individuals to assess their risk of impaired fasting glucose, especially given the lack of such models for the adult population across multiple centers in China. Our study is currently underway to create and validate personalized nomograms for predicting IFG in a diverse cohort of Chinese adults across 32 sites and 11 cities. The goal of this research is to provide clinical professionals with a reliable tool for accurately identifying individuals at risk and conducting timely screenings. The model is designed to be cost-effective and easily accessible for widespread use.

## Materials and methods

### Study design and participants

**Figure 1:**
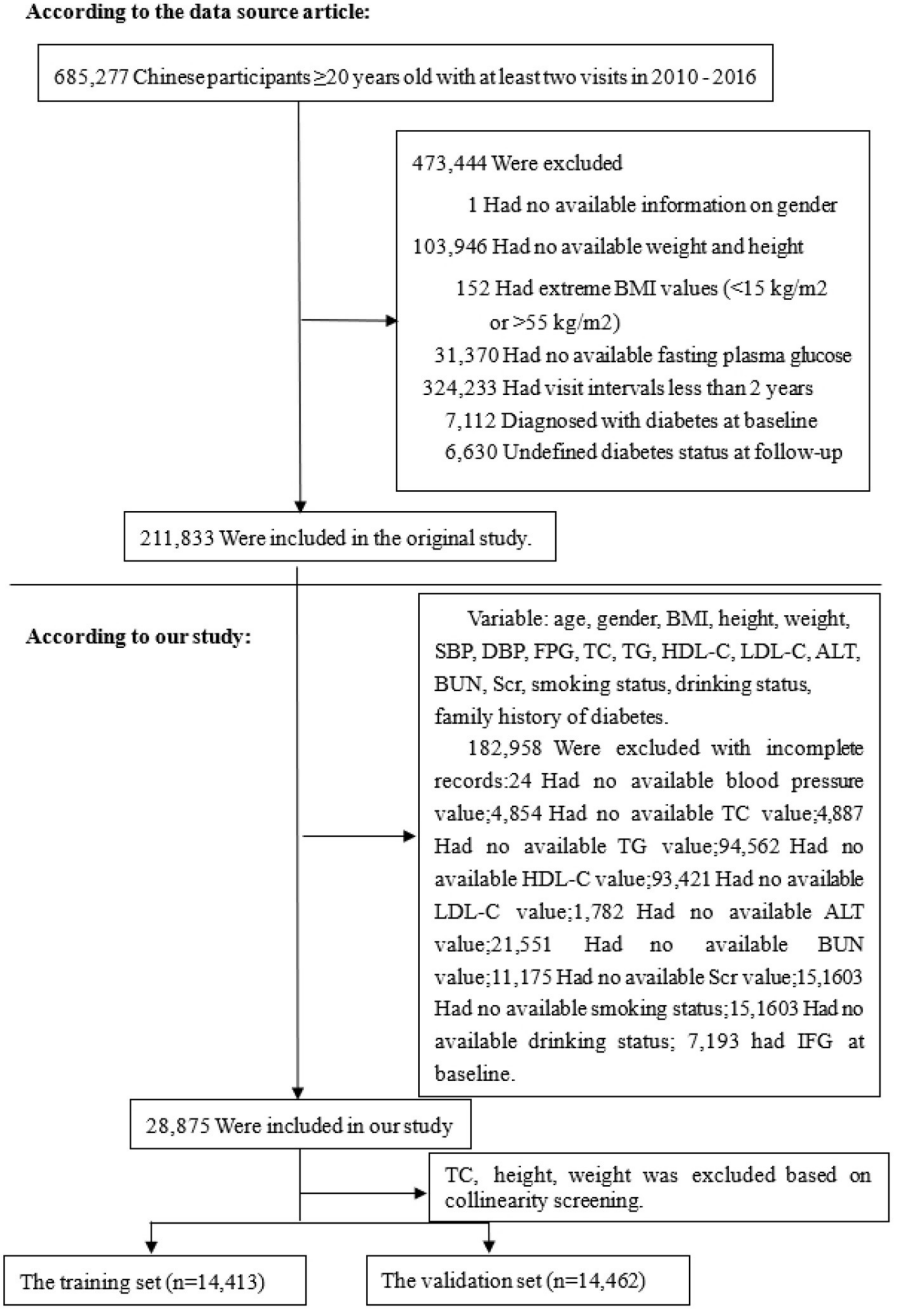
Flowchart of study participants.

Based on the data from the China Rich Healthcare Group's database, this retrospective cohort study was conducted with a 5-year follow-up period focusing on IFG as the dependent variable (17). IFG was categorized into two groups: non-IFG and IFG. The data utilized in this study was obtained from the publicly accessible, non-profit database DATADRYAD (http://www.DatadRyad.org) established by the Rich Healthcare Group. The data, sourced from Chen et al. (17), is publicly available and originates from the study “Association of body mass index and age with incident diabetes in Chinese adults: a population-based cohort study.” It can be accessed in the Dryad Digital Repository at http://dx.doi.org/10.1136/bmjopen-2018-021768. A total of 685,277 participants aged 20 and above who underwent at least two standard health examinations between 2010 and 2016 were included in the initial study. In the baseline study, demographic and clinical variables including age, gender, smoking status, alcohol consumption, family history of diabetes, body mass index (BMI), systolic and diastolic blood pressures (SBP and DBP), fasting plasma glucose (FPG), total cholesterol (TC), triglycerides (TG), low-density and high-density lipoprotein cholesterol (LDL-C and HDL-C), serum urea nitrogen (BUN), serum creatinine (Scr), alanine aminotransferase (ALT), years of follow-up, and censor of IFG at follow-up were collected. The initial study employed the following criteria to exclude participants: (1) absence of weight, height, and gender data; (2) BMI falling outside the range of 15–55 kg/m^2^; (3) visit intervals shorter than 2 years; (4) lack of FPG levels; and (5) presence of diabetes or indeterminate diabetes status. The final sample size comprised 211,833 participants. Furthermore, individuals without baseline data essential for assessing the risk of developing IFG were also excluded. The participant selection process is depicted in Figure 1. Our analysis included a total of 28,875 subjects. The data collected is anonymous, and the Rich Healthcare Group Review Board waived the need for informed consent due to the observational design of the study.

### Variable measurement

At the health check center, every participant completed an individual questionnaire inquiring about demographics, lifestyle, medical history, and family history of chronic diseases. An experienced staff member conducted the initial examination, which involved anthropometric measurements and laboratory biochemical testing. Participants were weighed and measured with a precision of 0.1 kg without wearing shoes. Blood pressure was measured using a standard mercury sphygmomanometer, and BMI was calculated by dividing weight by height squared. During the study, fasting venous blood samples were obtained from participants after a minimum fasting period of 10 h. Plasma glucose levels were measured using glucose oxidase, and a Beckman 5800 autoanalyzer was employed to evaluate FPG, TC, TG, LDL-C, HDL-C, BUN, Scr, and ALT. Standardized conditions and consistent procedures were maintained throughout the data collection process, with laboratory methods rigorously standardized through extensive quality checks at both internal and external levels.

### Definition of outcome

According to the Chinese Type II Diabetes Prevention and Control Guidelines (2017 Edition), FBG between 6.1 and 6.9 mmol/L was consider as IFG (18). Patients were censored either at the time of the diagnosis or at the last visit, whichever comes first.

### Statistical analysis

The training and validation sets were randomly allocated to all eligible participants. Through collinearity screening, variables displaying significant interference were excluded. The variance inflation factor (VIF) was calculated for each variable, and those with a VIF >5 were removed to address severe multicollinearity (19), which can significantly decrease the model's statistical stability and predictive accuracy (Supplementary Table S2).

Normally distributed continuous variables were reported as means and standard deviations, skewed variables as medians, and categorical variables as frequencies or percentages. The study utilized student *t*-tests to assess disparities between the training and validation sets for normally distributed continuous variables, Wilcoxon rank-sum tests for non-normally distributed continuous variables, and chi-square tests for categorical variables. Standardized differences of < 0.10 for a given covariate indicate a relatively small imbalance (20). Logit regression models were employed to ascertain the significance of each variable in identifying independent risk factors associated with IFG.

This study compared four distinct risk prediction models with the aim of developing a straightforward and dependable model for risk prediction. Initially, a comprehensive model incorporating all available risk factors (full model) was constructed, followed by a bidirectional stepwise selection process guided by the Akaike information criterion to streamline the model (stepwise model). Subsequently, the multivariable fractional polynomials algorithm was employed to identify crucial variables through a backward elimination process, culminating in a highly practical model (MFP model). Finally, the model underwent initial variable screening utilizing the Least Absolute Shrinkage and Selection Operator, a method known for its ability to streamline high-dimensional data and identify key predictors (21).

LASSO was chosen for its reduced number of variables and robust predictive capabilities during our comprehensive analysis (22), offering clinical interpretability and practical value compared to more complex models by significantly simplifying model complexity. ROC curves were generated, and the AUC along with 95% CI were calculated for both the training and validation datasets. The LASSO method was utilized to construct a nomogram that converts the regression coefficients obtained from a multivariate logistic regression analysis into a scoring system ranging (23). The variable with the largest absolute β coefficient was allocated 100 points. Subsequently, the cumulative points for all independent variables were computed and transformed into predicted probabilities of developing IFG. Each patient's nomogram score denoted their position within the predictive model, which was then juxtaposed with the observed 5-year incidence of IFG risk deciles in the training dataset.

To evaluate the agreement between the predicted and the actual 5-year risk of incident IFG, the Hosmer–Lemeshow test was used across deciles of predicted risk. The calibration of the model was further examined using a calibration plot. To address potential overfitting and assess model stability, 500 bootstrap resamples were applied to derive bias-corrected AUC estimates and enhance the credibility of the nomogram. To further evaluate the clinical utility of the LASSO regression model, we performed decision curve analysis (DCA) in the training sets. Standardized net benefit was plotted against high-risk thresholds to assess and visualize the added value of the LASSO nomogram in predicting 5-year IFG risk.

External validation was performed using two independent cohorts: (1) 18,618 individuals from routine health examinations at the Health Management Department of Shunde Hospital, Southern Medical University (January 2021–September 2022), and (2) 2,038 participants from NHANES 2017–2018. For both datasets, individuals with IFG at baseline were excluded. Missing values were imputed using the “mice” package in R.

All statistical analyses were performed using R software, version 4.3 (The R Foundation for Statistical Computing, http://www.R-project.org/). All tests were two-tailed, and a *P*-value of < 0.05 was considered statistically significant.

## Results

### Baseline characteristics of study participants

A total of 28,875 qualified participants were included in this study, with men comprising 64.27% and women 35.73%. The average age was 42.54 ± 12.37 years. During a median follow-up period of 2.96 years (range: 2.001–4.999 years), 948 participants were diagnosed with IFG. The average BMI was 23.44 ± 3.26 kg/m^2^; the mean SBP and DBP were 119.26 ± 15.57 and 74.64 ± 10.41 mmHg, respectively. The mean FPG level was 4.91 ± 0.54 mmol/L, with HDL-C and LDL-C averaging 1.34 ± 0.30 and 2.74 ± 0.68 mmol/L. Baseline levels of BUN and Scr were 4.68 ± 1.15 mmol/L and 72.14 ± 15.19 μmol/L, respectively. The mean follow-up duration was 2.99 ± 0.85 years. We summarize the demographic, clinical, and anthropometric characteristics of participants, showing no significant differences between the training set (*n* = 14,413) and validation set (*n* = 14,462) at baseline (all *P* > 0.05; Table 1). We also categorized participants based on IFG status (Supplementary Table S1).

**Table 1:** Baseline characteristics of the training and validation sets.

**Characteristic**	**All participants**	**Training set**	**Validation set**	**Standardized difference**	***P*-value^a^**
Participants	28,875	14,413	14,462		
Age (year)	42.54 ± 12.37	42.60 ± 12.38	42.48 ± 12.36	0.01	0.402
**Gender**					
Male	18,557 (64.27%)	9,258 (64.23%)	9,299 (64.30%)	0.00	0.917
Female	10,318 (35.73%)	5,155 (35.77%)	5,163 (35.70%)		
BMI (kg/m^2^)	23.44 ± 3.26	23.44 ± 3.25	23.44 ± 3.28	0.00	0.946
SBP (mmHg)	119.26 ± 15.57	119.25 ± 15.52	119.27 ± 15.62	0.00	0.928
DBP (mmHg)	74.64 ± 10.41	74.65 ± 10.40	74.63 ± 10.43	0.00	0.883
FPG (mmol/L)	4.91 ± 0.54	4.91 ± 0.55	4.91 ± 0.54	0.01	0.361
TG (mmol/L)	1.42 ± 1.04	1.41 ± 1.01	1.43 ± 1.07	0.02	0.185
HDL-C (mmol/L)	1.34 ± 0.30	1.34 ± 0.30	1.35 ± 0.30	0.01	0.356
LDL-C (mmol/L)	2.74 ± 0.68	2.74 ± 0.69	2.74 ± 0.68	0.00	0.730
ALT (U/L)	25.03 ± 21.08	25.10 ± 21.54	24.96 ± 20.60	0.01	0.586
BUN (mmol/L)	4.68 ± 1.15	4.69 ± 1.16	4.68 ± 1.14	0.01	0.295
Scr (umol/L)	72.14 ± 15.19	72.17 ± 15.25	72.12 ± 15.13	0.00	0.769
**Smoking status**					
Ever/Current	6,861 (23.76%)	3,419 (23.72%)	3,442 (23.80%)	0.00	0.886
Never	22,014 (76.24%)	10,994 (76.28%)	11,020 (76.20%)		
**Drinking status**					
Ever/Current	5,560 (19.26%)	2,748 (19.07%)	2,812 (19.44%)	0.01	0.424
Never	23,315 (80.74%)	11,665 (80.93%)	11,650 (80.56%)		
**Family history**					
No	27,308 (94.57%)	13,621 (94.50%)	13,687 (94.64%)	0.01	0.628
Yes	1,567 (5.43%)	792 (5.50%)	775 (5.36%)		

^a^Welch Two Sample t-test; Pearson's Chi-squared test.

Values are n (%) or mean ± SD.

BMI, body mass index; SBP, systolic blood pressure; DBP, diastolic blood pressure; FPG; fasting plasma glucose; TG, triglyceride; HDL-C, high density lipoprotein cholesterol; LDL-C, low density lipid cholesterol; ALT, alanine aminotransferase; BUN, blood urea nitrogen; Scr, serum creatinine; Family history, family history of diabetes.

### Identification of independent risk factors for IFG

Univariate and multivariate logistic regression analyses were conducted to identify risk factors for incident IFG. In univariate analysis, all examined variables were significant predictors of IFG (all *P* < 0.05). After adjustment in the multivariate model, age (OR = 1.03), BMI (OR = 1.08), SBP (OR = 1.00), DBP (OR = 1.01), FPG (OR = 5.85), TG (OR = 1.21), HDL-C (OR = 0.67), ALT (OR = 1.00), Scr (OR = 0.98), and a family history of diabetes (OR = 1.78) remained significantly associated with the risk of IFG (all *P* < 0.05). In contrast, gender, LDL-C, BUN, smoking status, and alcohol consumption were not significantly associated with IFG (all *P* > 0.05; Table 2).

**Table 2:** Risk predictors for incident diabetes in the univariate and multivariate analysis.

**Variable**	**Univariate [OR (95% CI), *P*]**	**Multivariate [OR (95% CI), *P*]**
Age (year)	1.05 (1.04–1.06), < 0.05	1.03 (1.02–1.04), < 0.05
**Gender**		
Male	Ref	Ref
Female	0.62 (0.5–0.76), < 0.05	0.82 (0.59–1.12), 0.217
BMI (kg/m^2^)	1.19 (1.16–1.22), < 0.05	1.08 (1.04–1.12), < 0.05
SBP (mmHg)	1.04 (1.03–1.04), < 0.05	1.00 (1.00–1.01), < 0.05
DBP (mmHg)	1.05 (1.04–1.06), < 0.05	1.01 (1.00–1.02), 0.022
FPG (mmol/L)	7.4 (5.99–9.19), < 0.05	5.85 (4.19–8.16), < 0.05
TG (mmol/L)	1.41 (1.33–1.49), < 0.05	1.21 (1.11–1.30), < 0.05
HDL-C (mmol/L)	0.43 (0.31–0.6), < 0.05	0.67 (0.47–0.96), < 0.05
LDL-C (mmol/L)	1.49 (1.31–1.68), < 0.05	1.07 (0.93–1.23), 0.294
ALT (U/L)	1.01 (1–1.01), < 0.05	1.00 (1.00–1.01), < 0.05
BUN (mmol/L)	1.23 (1.14–1.32), < 0.05	1.06 (0.97–1.15), 0.148
Scr (μmol/L)	1.01 (1–1.01), < 0.05	0.98 (0.97–0.99), < 0.05
**Smoking status**		
Ever/Current	Ref	Ref
Never	0.66 (0.55–0.81), < 0.05	0.975 (0.77–1.22), 0.834
**Drinking status**		
Ever/Current	Ref	Ref
Never	0.76 (0.61–0.94), < 0.05	0.97 (0.76–1.24), 0.846
**Family history**		
No	Ref	Ref
Yes	1.61 (1.14–2.22), < 0.05	1.78 (1.25–2.55), < 0.05

BMI, body mass index; SBP, systolic blood pressure; DBP, diastolic blood pressure; FPG; fasting plasma glucose; TG, triglyceride; HDL-C, high-density lipoprotein cholesterol; LDL-C, low-density lipid cholesterol; ALT, alanine aminotransferase; BUN, blood urea nitrogen; Scr, serum creatinine; Family history, family history of diabetes. OR, Odds ratio; CI, confidence interval; Ref, reference.

### Development and validation of IFG risk prediction models

**Figure 2:**
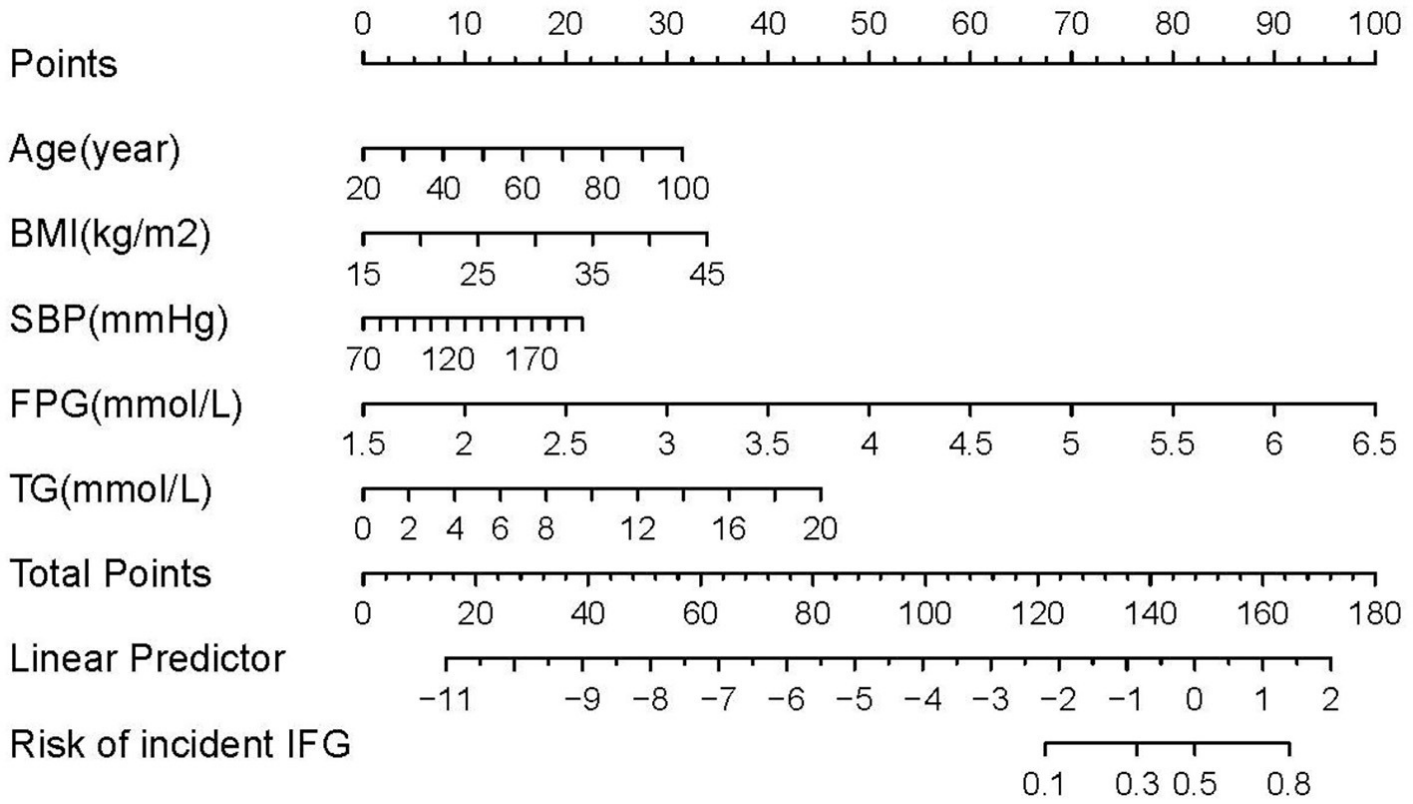
Nomogram to predict the risk of IFG for Chinese adults. The patient's score for each risk predictor is plotted on the appropriate scale. The patient's score for each risk predictor is plotted on the appropriate scale and vertical lines are drawn from that value to the top Points scale to obtain the corresponding scores. All scores are summed to obtain the total points score. The total points score is plotted on the bottom Total Points scale. The corresponding value shows the predicted probability of incident IFG.

We constructed four risk prediction models—the full model, stepwise model, MFP model, and LASSO model (Supplementary Figure S1). Given its simplicity and robust predictive performance, the LASSO model was selected for nomogram development, identifying five key predictors: age, BMI, SBP, FPG, and TG (Figure 2). The 5-year IFG risk can be estimated using the following formula: −17.71490 + 0.03257 × age (years) + 0.09353 × BMI (kg/m^2^) + 0.01376 × SBP (mmHg) + 1.6520 × FPG (mmol/L) + 0.18660 × TG (mmol/L). The predictive performance details are presented in Table 3, with the LASSO model demonstrating comparable accuracy to other models.

**Table 3:** Prediction performance of LASSO, MFP, full and stepwise model for the risk of diabetes.

	**Training**				**Validation**			
	**LASSO**	**Full**	**Stepwise**	**MFP**	**LASSO**	**Full**	**Stepwise**	**MFP**
AUC	0.8167	0.8249	0.8244	0.8191	0.8155	0.8214	0.8212	0.8174
95% CI Lower	0.7983	0.8071	0.8066	0.8009	0.7967	0.803	0.8027	0.7987
95% CI Upper	0.8352	0.8427	0.8422	0.8372	0.8342	0.8399	0.8397	0.8361
Best threshold	0.0368	0.0412	0.0372	0.0363	0.0301	0.0332	0.032	0.0354
Specificity, %	75.82	78.66	76.25	75.43	70.93	74.04	73.19	75.17
Sensitivity, %	75.37	72.63	74.95	75.16	77.17	76.11	76.74	74
Accuracy, %	75.81	78.46	76.21	75.42	71.14	74.1	73.3	75.13
PPV, %	9.6	10.39	9.71	9.44	8.24	9.02	8.82	9.16
NPV, %	98.91	98.83	98.89	98.89	98.92	98.92	98.94	98.84
PLR	3.12	3.4	3.16	3.06	2.65	2.93	2.86	2.98
NLR	0.32	0.35	0.33	0.33	0.32	0.32	0.32	0.35
DOR	9.6	9.78	9.61	9.29	8.25	9.08	9.01	8.62

LASSO, least absolute shrinkage and selection operator; MFP, multivariable fractional polynomials; AUC, area under curve; CI, confidence interval; PPV, positive predictive value; NPV, negative predictive value; PLR, positive likelihood ratio; NLR, negative likelihood ratio; DOR, diagnostic odds ratio.

### Prediction performance and clinical utility of the LASSO model

**Figure 3:**
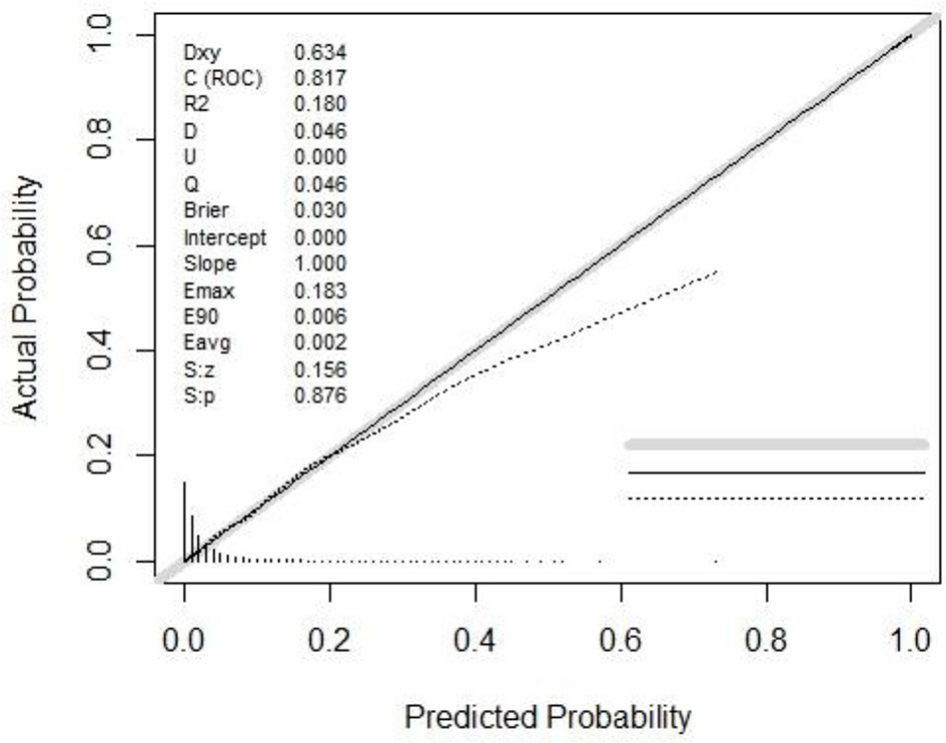
Calibration plot for LASSO regression model in predicting IFG.

The AUC values of the LASSO model for the training and validation sets were 0.8167 and 0.8155, respectively (Table 3). Additionally, bootstrap validation demonstrated consistent AUC values for the prediction nomogram. The predictive performance of the full, stepwise, and MFP models is also presented in Table 3 and Supplementary Figure S2. Calibration of the LASSO model was assessed using both a calibration plot (Figure 3) and the Hosmer–Lemeshow test. The calibration plot showed that the predicted probabilities closely matched the actual incidence of IFG in the training set, indicating good overall calibration. In addition, the Hosmer–Lemeshow test assessed the agreement between predicted and observed risks across deciles of predicted risk, demonstrating no statistically significant difference (*P* > 0.05), which further supports the reliable calibration of the nomogram (Supplementary Figure S3).

**Figure 4:**
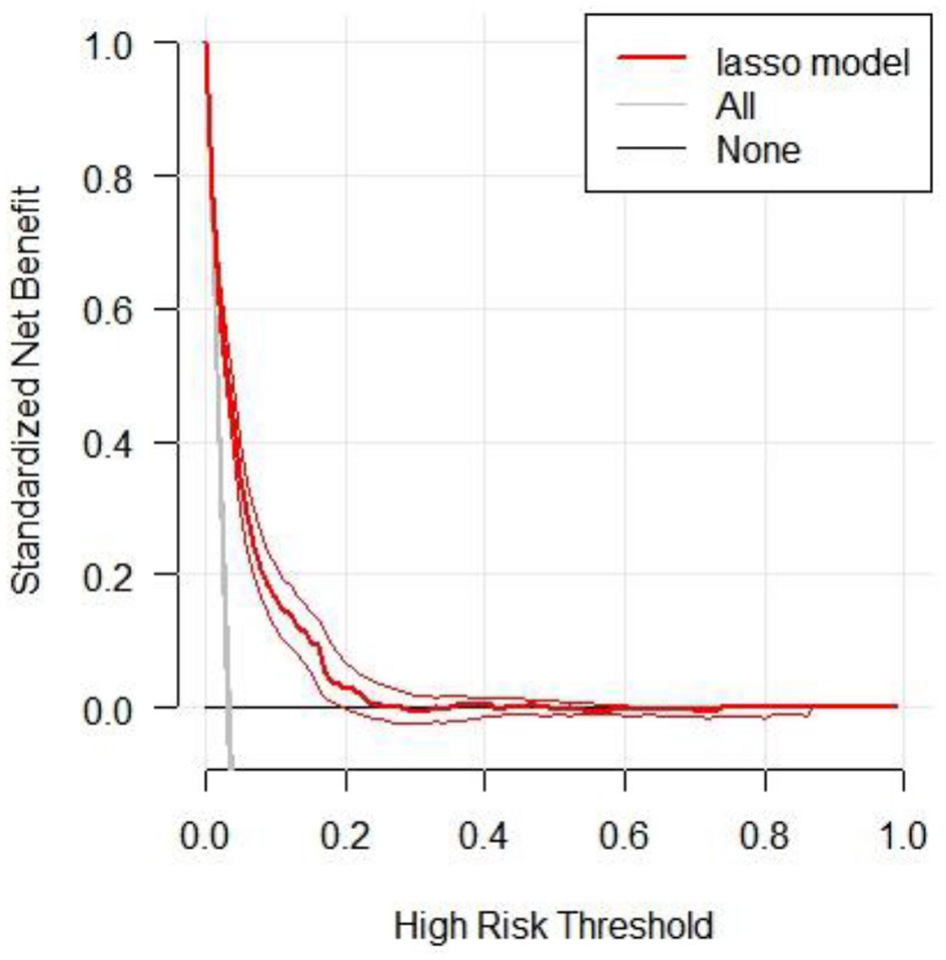
The decision curve analysis of the LASSO model for 5-year IFG risk in the training cohort. The black line represents the net benefit when none of the participants are considered to develop IFG, while the light gray line represents the net benefit when all participants are considered to develop IFG. The area between the “no treatment line” (black line) and “all treatment line” (light gray line) in the model curve indicates the clinical utility of the model. The farther the model curve is from the black and light gray lines, the better the clinical use of the nomogram.

We conducted decision curve analysis (DCA) to evaluate the clinical utility of the LASSO model (Figure 4). The DCA results demonstrated that across a range of high-risk thresholds (0.0–1.0), the standardized net benefit curve of the LASSO model consistently exceeded the baseline strategies of “no treatment line” (black line) and “all treatment line” (light gray line). This indicates that clinicians would achieve clinical benefit when using the LASSO model for decision-making within the risk prediction threshold range. The area between the model curve and the two baseline lines represents the clinical utility of the model, with greater distance between the model curve and baseline lines indicating higher clinical value of the nomogram. Furthermore, Supplementary Figure S4 provided a comparative analysis of decision curves for four models (full model, stepwise model, MFP model, and LASSO model) in both training and validation cohorts. The results indicated that despite using the fewest predictors, the LASSO model demonstrated clinical utility comparable to more complex models. This further confirms the rationale for selecting the LASSO model to develop the final risk prediction nomogram, which achieves maximum clinical utility and simplicity while maintaining high predictive performance.

### External validation

External validation was performed using two independent datasets. The first cohort included 18,618 participants from the Health Management Department of Shunde Hospital, Southern Medical University. The second cohort comprised 2,038 participants from the NHANES 2017–2018 survey. The AUC values for external validation were 0.9665 for the Shunde Hospital cohort and 0.9171 for the NHANES cohort (Supplementary Tables S3, S4). At the optimal threshold, the Shunde Hospital cohort showed a specificity of 88.83% and a sensitivity of 93.66%, while the NHANES cohort demonstrated a specificity of 81.48% and a sensitivity of 86.51%, indicating excellent predictive performance of the nomogram in both external populations.

### Sensitivity analysis

For the sensitivity analysis, individuals with IFG at baseline were first excluded from the overall population in the original study, resulting in 202,402 participants included for analysis. Multiple imputations were then performed for the remaining population with missing data on relevant variables. The LASSO model was applied to this imputed cohort, achieving an AUC of 0.8308. At the optimal threshold, the specificity and sensitivity were 73.76 and 78.14%, respectively (Supplementary Table S4).

## Discussion

This retrospective cohort study developed and validated a personalized nomogram for predicting 5-year IFG risk in Chinese adults using five readily available clinical parameters (age, BMI, SBP, FPG, and TG). The LASSO regression model demonstrated predictive performance through internal validation and was tested in the external validation dataset. Decision curve analysis evaluated the model's clinical applicability at different risk thresholds, providing clinicians with a potential risk assessment tool.

Numerous studies have indicated that IFG plays a significant role as a transitional phase between normal health and the onset of DM. IFG is an independent risk factor for the development of DM (24). Effective prevention and management of DM have been shown to hinge upon the reduction of IFG rates (25). Early identification and intervention for DM have also proven to be effective strategies in the management of the disease. Compared to the clinical and laboratory parameter nomogram model developed by Wang et al. (16) based on over 4,000 individuals, our model differs in terms of the number of predictive factors and model complexity. The model includes six predictive factors: age, systolic blood pressure, BMI, albumin, urea, and triglycerides. Its AUC values are 0.783 for the training set and 0.7891 for the validation set. Our model demonstrates certain potential for clinical application in predictive performance. Compared to another IFG prediction model developed using the extreme gradient boosting (XGBoost) algorithm (26), our study explores a different approach to model interpretability. This model achieved an AUC value of 0.7391 in the validation set and included key predictive factors such as SBP, waist circumference, fatty liver, and serum creatinine. While machine learning algorithms like XGBoost offer powerful predictive capabilities, they often present challenges in clinical interpretation due to their complex underlying mechanisms. Our nomogram addresses this challenge by providing a visually intuitive representation that facilitates understanding for both healthcare professionals and patients. Our study employed both internal and external validation to thoroughly assess its generalizability, drawing parallels with Byeon's model (27), which achieved an AUC of 0.751 in predicting IFG risk among non-diabetic individuals in South Korea. To further evaluate the clinical applicability of our model, we conducted DCA across a wide range of risk thresholds. While many existing models have not extensively explored this analytical approach, our research aimed to provide a more nuanced understanding of the model's practical utility.

The IFG predictive nomogram model developed in this research demonstrates good clinical practicality, particularly in primary healthcare and resource-limited settings. The model relies solely on five routine clinical parameters, requiring no additional complex tests, a characteristic that enables its flexible application across different medical resource environments. In primary healthcare and community health service centers, this model can serve as a supplementary tool to routine physical examinations, enabling large-scale screening and the timely identification of individuals at high risk for IFG. It is especially practical in resource-limited rural areas, where risk assessment can be completed using only basic diagnostic equipment. In urban general hospitals, the model can be integrated into the routine assessment processes of health management centers and embedded within electronic health record systems to achieve automated risk evaluation, providing a basis for physicians to develop personalized health management plans. In the field of public health, this model can be used for population-level monitoring of IFG risk, offering scientific evidence for policy-making. With its characteristics of simplicity, cost-effectiveness, and ease of operation, this model is expected to become an effective tool for early identification and intervention of prediabetes in primary healthcare institutions.

Within the model, five specific risk factors were identified as significantly correlated with IFG, a finding consistent with prior research demonstrating these factors as key determinants of IFG (28, 29). A s individuals age, the risk of developing diabetes increases due to age-related changes in pancreatic β cells, including reduced glucose sensitivity and insulin secretion. This age-related glucose intolerance is often associated with insulin resistance and β-cell dysfunction (30). Additionally, aging human pancreatic islets may experience a decrease in mitochondrial DNA copy number, further impairing insulin secretion (31). Obesity is known to substantially elevate the likelihood of developing a range of metabolic disorders, particularly affecting the function of pancreatic β-cells by promoting excessive fat deposition in the liver and pancreas. Research has demonstrated a correlation between obesity and heightened levels of pancreatic fat accumulation (32). Additionally, regarding blood pressure, in the study by Sasaki et al. (33), it is established that the rate of hypertension significantly increases from normal fasting glucose to isolated IFG, indicative of a direct association between IFG and elevated hypertension risk. Our nomogram indicates that individuals with elevated FPG levels exhibit a greater propensity for IFG, with FPG emerging as a distinct risk factor in this context. This relationship is likely attributable to FPG's substantial impact on insulin responsiveness and sensitivity (34). Triglycerides play a crucial role in lipid storage and metabolic regulation within adipose tissue (35). The presence of excessive adipose tissue can significantly worsen insulin resistance by generating proinflammatory cytokines and lipid metabolites that induce insulin resistance (36). This highlights the complex interplay between adiposity, inflammation, and metabolic dysfunction, ultimately contributing to the development of IFG. As such, the inclusion of the five risk predictors in our models is justified.

### Strengths and limitations of this study

The strengths of our study include a large sample size derived from a diverse participant pool across multiple centers, the selection of the LASSO model for its simplicity and predictive performance in developing four predictive models for clinical feasibility, the ability for clinicians to efficiently assess an individual's risk of impaired fasting glucose using a formula for risk prediction, the verification of findings through internal and external validations, and the mitigation of selection and information biases through a retrospective cohort design.

Several limitations should be acknowledged in our study. First, this research is primarily a secondary retrospective analysis, with inherent constraints in the original dataset. The data lacks comprehensive information on variables such as waist-to-hip ratio, detailed medical history, and comprehensive lifestyle factors—all of which could potentially influence IFG development. Second, although methodologically sound, our utilization of multiple imputation techniques to address missing data may potentially introduce bias into the analysis, thereby affecting the precision of our estimates. Additionally, this study used data from a cohort study conducted during 2010–2016, and we acknowledge that these relatively older data may impose certain limitations, potentially not fully reflecting current trends in the non-communicable disease burden among the Chinese population. Given the limitations of current research, future studies should prioritize comprehensive prospective research, improved baseline measurements, and the integration of lifestyle and genetic factor assessments to enhance the accuracy and generalizability of models for predicting the risk of IFG.

## Conclusion

A personalized prediction nomogram was developed and validated to assess the 5-year risk of developing IFG among Chinese adults based on age, BMI, SBP, FPG, and TG levels. The nomogram exhibited excellent predictive accuracy during both training and validation phases, indicating its potential for generalizability. This tool aids clinicians in identifying individuals at high risk for IFG through a straightforward and dependable approach.

## Data Availability

The datasets presented in this study can be found in online repositories. The names of the repository/repositories and accession number(s) can be found below: The 'DATADRYAD' database (http://www.Datadryad.org) provides access to data.
