# Supplementary material for: A study on predicting impaired fasting glucose risk in Chinese adults based on individual characteristics
Source: Front Med (Lausanne). 2025 Jun 9;12:1584626. doi: 10.3389/fmed.2025.1584626 (PMC12183226; doi:10.3389/fmed.2025.1584626)

**Supplementary Material**

**A Study on Predicting Impaired Fasting Glucose Risk in Chinese Adults Based on Individual Characteristics**

**Online supplementary table 1.** Baseline characteristics for the training and validation sets by incident IFG status.

**Online supplementary table 2.** VIF Assessment of Predictor Variables and Collinearity Optimization Comparison.

**Online supplementary table 3.** Comparison of baseline characteristics among external validation sets (n=2) and the overall population of the original study.

**Online supplementary table 4.** Prediction performance comparison for IFG risk across external validation sets (n=2) and the overall population of the original study.

**Online supplementary figure 1.** Variable Selection Process in the LASSO Logistic Regression Model.

**Online supplementary figure 2.** Comparison of ROC Curves Among LASSO, Full, Stepwise, and MFP Models in Training and Validation Sets.

**Online supplementary figure 3.** Comparison between predicted and observed 5-year incidence of IFG of predicted IFG risk score for the training set in the nomogram.

**Online supplementary figure 4.** The decision curve analysis of the full model, stepwise model, MFP model and LASSO model for 5-year IFG risk in the training cohort (a) and validation cohort (b).

**Online supplementary figure 5.** ROC curves with confidence intervals for LASSO, Full, Stepwise, and MFP models (Training Set)

**Online supplementary figure 6.** ROC curves with confidence intervals for LASSO, Full, Stepwise, and MFP models (Validation Set)

**Online supplementary figure 7.** Decision Curve Analysis with Confidence Intervals for LASSO, Full, Stepwise, and MFP models (Training Set)

**Online supplementary figure 8.** Decision Curve Analysis with Confidence Intervals for LASSO, Full, Stepwise, and MFP models (Validation Set)

Online supplementary table 1. Baseline characteristics for the training and validation sets by incident IFG status.

| Characteristic |  | training set |  |  | validation set |  |
| --- | --- | --- | --- | --- | --- | --- |
|  | **Non-IFG** | **IFG** | **p-value^1^** | **Non-IFG** | **IFG** | **p-value** |
| Participants | **13938** | **475** |  | **13989** | **473** |  |
| Age (year) | **42.30 ± 12.26** | **51.26 ± 12.90** | **<0.001** | **42.19 ± 12.24** | **50.92 ± 12.89** | **<0.001** |
| Gender |  |  | **<0.001** |  |  | **<0.001** |
| Male | **8906 (63.90%)** | **352 (74.11%)** |  | **8946 (63.95%)** | **353 (74.63%)** |  |
| Female | **5032 (36.10%)** | **123 (25.89%)** |  | **5043 (36.05%)** | **120 (25.37%)** |  |
| BMI (kg/m2) | **23.37 ± 3.23** | **25.41 ± 3.16** | **<0.001** | **23.37 ± 3.26** | **25.41 ± 3.27** | **<0.001** |
| SBP (mmHg) | **118.93 ± 15.35** | **128.76 ± 17.42** | **<0.001** | **118.98 ± 15.51** | **127.80 ± 16.45** | **<0.001** |
| DBP (mmHg) | **74.46 ± 10.33** | **80.36 ± 10.79** | **<0.001** | **74.46 ± 10.39** | **79.72 ± 10.40** | **<0.001** |
| FPG (mmol/L) | **4.90 ± 0.54** | **5.38 ± 0.49** | **<0.001** | **4.89 ± 0.53** | **5.40 ± 0.49** | **<0.001** |
| TG (mmol/L) | **1.39 ± 0.99** | **2.04 ± 1.35** | **<0.001** | **1.41 ± 1.05** | **1.95 ± 1.43** | **<0.001** |
| HDL-C (mmol/L) | **1.35 ± 0.30** | **1.28 ± 0.29** | **<0.001** | **1.35 ± 0.30** | **1.28 ± 0.30** | **<0.001** |
| LDL-C (mmol/L) | **2.73 ± 0.69** | **2.94 ± 0.66** | **<0.001** | **2.74 ± 0.68** | **2.91 ± 0.74** | **<0.001** |
| ALT (U/L) | **24.90 ± 21.60** | **30.97 ± 18.81** | **<0.001** | **24.75 ± 20.39** | **31.27 ± 25.45** | **<0.001** |
| BUN (mmol/L) | **4.68 ± 1.16** | **4.98 ± 1.20** | **<0.001** | **4.67 ± 1.13** | **4.98 ± 1.18** | **<0.001** |
| Scr (umol/L) | **72.12 ± 15.26** | **73.54 ± 15.08** | **0.044** | **72.07 ± 15.16** | **73.49 ± 14.13** | **0.032** |
| Smoking status |  |  | **<0.001** |  |  | **0.025** |
| Ever/Current | **3269 (23.45%)** | **150 (31.58%)** |  | **3276 (23.42%)** | **166 (35.10%)** |  |
| Never | **10669 (76.55%)** | **325 (68.42%)** |  | **10713 (76.58%)** | **307 (64.90%)** |  |
| Drinking status |  |  | **0.013** |  |  | **0.029** |
| Ever/Current | **2636 (18.91%)** | **112 (23.58%)** |  | **2701 (19.31%)** | **111 (23.47%)** |  |
| Never | **11302 (81.09%)** | **363 (76.42%)** |  | **11288 (80.69%)** | **362 (76.53%)** |  |
| Family history |  |  | **0.006** |  |  | **0.035** |
| No | **13186 (94.60%)** | **435 (91.58%)** |  | **13250 (94.72%)** | **437 (92.39%)** |  |
| Yes | **752 (5.40%)** | **40 (8.42%)** |  | **739 (5.28%)** | **36 (7.61%)** |  |

^1^Welch Two Sample t-test; Pearson's Chi-squared test

Values are n (%) or mean ± SD.

BMI, Body mass index; SBP, Systolic blood pressure; DBP, Diastolic blood pressure; FPG; Fasting plasma glucose; TG, Triglyceride; HDL-C, High density lipoprotein cholesterol; LDL-C, Low density lipid cholesterol; ALT, Alanine aminotransferase; BUN, Blood urea nitrogen; Scr, Serum creatinine; Family history, Family history of diabetes.

Online supplementary table 2. VIF Assessment of Predictor Variables and Collinearity Optimization Comparison.

| Characteristic | Original Model | | Reduced Collinearity Model | |
| --- | --- | --- | --- | --- |
|  | VIF value | Collinearity Assessment | VIF value | Collinearity Assessment |
| Weight height ratio | 5624.564 | Severe multicollinearity | / | / |
| Weight | 2061.262 | Severe multicollinearity | / | / |
| BMI | 1268.703 | Severe multicollinearity | 1.495649471 | Acceptable Range |
| Height | 51.33729 | Severe multicollinearity | / | / |
| Cholesterol | 7.049051 | Collinearity exists | / | / |
| LDL | 5.752954 | Collinearity exists | 1.129760426 | Acceptable Range |
| GENDER | 3.52653 | Acceptable Range | 2.629231363 | Acceptable Range |
| CCR | 2.181228 | Acceptable Range | 2.164178439 | Acceptable Range |
| SBP | 2.044472 | Acceptable Range | 2.036675921 | Acceptable Range |
| DBP | 1.944795 | Acceptable Range | 1.939847819 | Acceptable Range |
| Triglyceride | 1.778495 | Acceptable Range | 1.290778205 | Acceptable Range |
| HDLc | 1.576861 | Acceptable Range | 1.232587658 | Acceptable Range |
| Age | 1.348202 | Acceptable Range | 1.263399197 | Acceptable Range |
| SMOKING | 1.281421 | Acceptable Range | 1.275751029 | Acceptable Range |
| ALT | 1.24685 | Acceptable Range | 1.237951321 | Acceptable Range |
| BUN | 1.178169 | Acceptable Range | 1.177374916 | Acceptable Range |
| Drinking | 1.147782 | Acceptable Range | 1.146834188 | Acceptable Range |
| FPG | 1.065103 | Acceptable Range | 1.063152974 | Acceptable Range |
| Family history of diabetes | 1.015751 | Acceptable Range | 1.015299021 | Acceptable Range |

Excluded Variables: Weight height ratio, Weight, Height, Cholesterol.

Retained Variables: BMI, LDL, GENDER, CCR, SBP, DBP, Triglyceride, HDLc, Age, SMOKING, ALT, BUN, Drinking, FPG, Family history of diabetes.

Online supplementary table 3. Comparison of baseline characteristics among external validation sets (n=2) and the overall population of the original study.

| Characteristic | External validation set 1 | External validation set 2 | The overall population | P value |
| --- | --- | --- | --- | --- |
| Participants | 18,618 | 2,038 | 202,402 |  |
| Age (year), Mean (SD) | 41.96 (12.67) | 49.38 (17.77) | 41.57 (12.36) | <0.001 |
| BMI (kg/m2), Mean (SD) | 23.76 (3.56) | 29.24 (7.20) | 23.12 (3.29) | <0.001 |
| SBP (mmHg), Mean (SD) | 122.03 (16.27) | 124.99 (19.78) | 118.51 (16.07) | <0.001 |
| FPG (mmol/L), Mean (SD) | 5.34 (0.53) | 5.66 (0.58) | 4.86 (0.55) | <0.001 |
| TG (mmol/L), Mean (SD) | 1.55 (1.37) | 1.37 (1.08) | 1.31 (0.98) | <0.001 |
| IFG, n (%) | 1,624 (8.72%) | 467(22.91%) | 6,456 (3.19%) | <0.001 |

Values are n (%) or mean ± SD

External validation set 1: Data from individuals undergoing routine health examinations at the Health Management Department of Shunde Hospital, Southern Medical University (January 2021 to September 2022)

External validation set 2:data from NHANES 2017-2018

BMI, Body mass index; SBP, Systolic blood pressure; FPG; Fasting plasma glucose; TG, Triglyceride; NHANES; National Health and Nutrition Examination Survey.

Online supplementary table 4. Prediction performance comparison for IFG risk across external validation sets (n=2) and the overall population of the original study.

|  | External validation set 1  (n=18,618) | External validation set 2  (n=2,038) | The overall population  (n=202,402) |
| --- | --- | --- | --- |
| AUC | 0.9665 | 0.9171 | 0.8308 |
| 95% CI |  |  |  |
| Lower | 0.9635 | 0.9042 | 0.8259 |
| Upper | 0.9696 | 0.9301 | 0.8358 |
| Best threshold | 0.1106 | 0.2071 | 0.0282 |
| Specificity, % | 88.83 | 81.48 | 73.76 |
| Sensitivity, % | 93.66 | 86.51 | 78.14 |
| Accuracy, % | 89.25 | 82.63 | 73.9 |
| PPV, % | 44.47 | 58.13 | 8.94 |
| NPV, % | 99.32 | 95.31 | 99.03 |
| PLR | 8.38 | 4.67 | 2.98 |
| NLR | 0.07 | 0.17 | 0.3 |
| DOR | 117.38 | 28.21 | 10.05 |

AUC, Area under curve; CI, Confidence interval; PPV, Positive predictive value; NPV, Negative predictive value; PLR, Positive likelihood ratio; NLR, Negative likelihood ratio; DOR, Diagnostic odds ratio. External validation set 1: Data from individuals undergoing routine health examinations at the Health Management Department of Shunde Hospital, Southern Medical University (January 2021 to September 2022); External validation set 2:data from NHANES 2017-2018

A


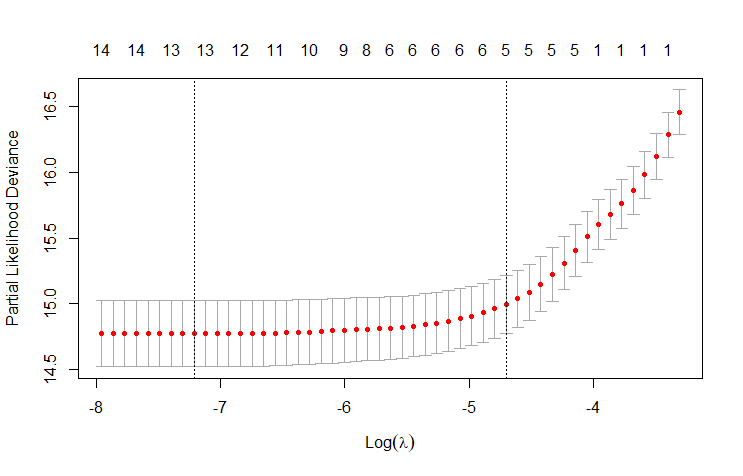


B


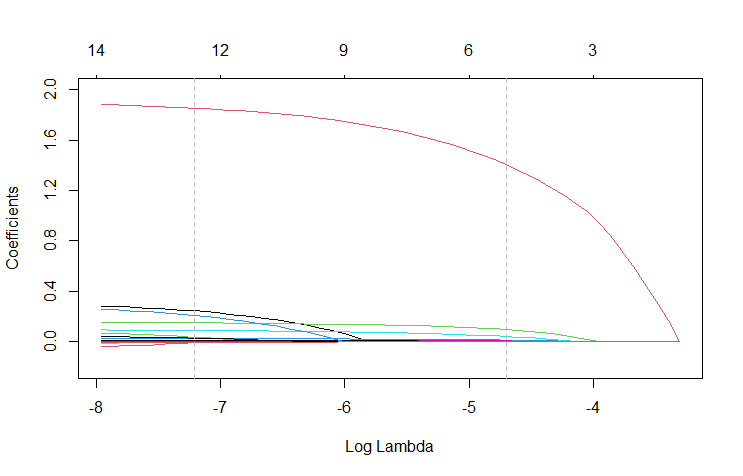


Online supplementary figure 1. Variable Selection Process in the LASSO Logistic Regression Model.

Risk predictors selection using the LASSO logistic regression model. (A) Optimal predictor (lambda) selection in the LASSO model with tenfold cross validation by minimum criteria. The area under the receiver operation characteristic curve was plotted versus log (lambda). Dotted vertical lines were drawn at the optimal values by using the minimum criteria and the 1 SE of the minimum criteria; (B) LASSO coefficient profiles of the 15 predictors. A coefficient profile plot was developed against the log (lambda) sequence. Vertical line was drawn at the value selected with tenfold cross validation, where optimal lambda resulted in 5 predictors with nonzero coefficients (lambda = 0.009).

A


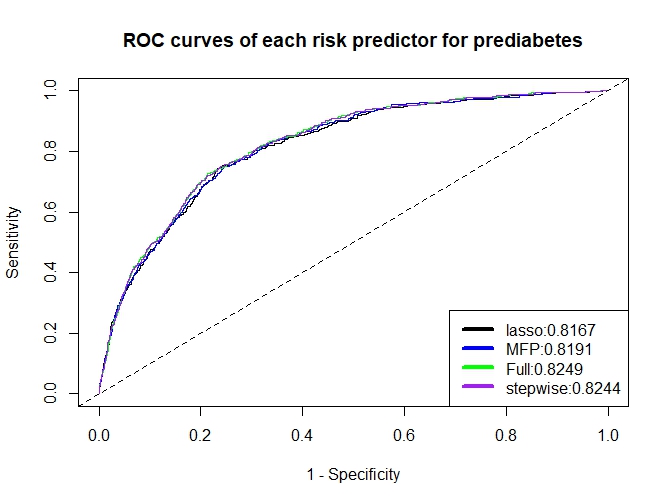


B


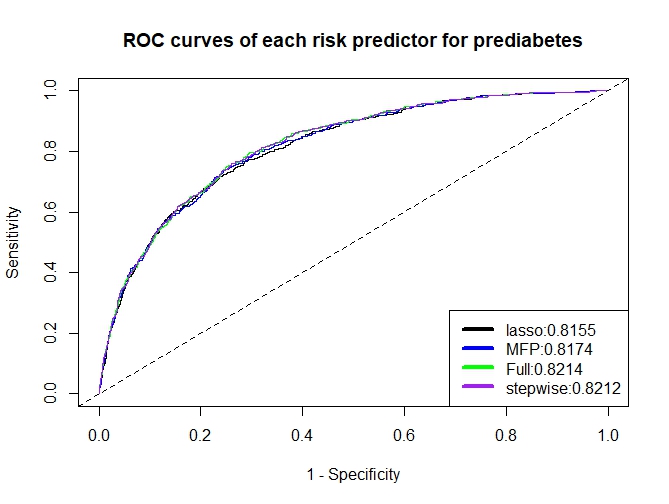


Online supplementary figure 2. Comparison of ROC Curves Among LASSO, Full, Stepwise, and MFP Models in Training and Validation Sets. The ROC curves of the LASSO model, full model, stepwise model and MFP model in the training set (A) and validation set (B).


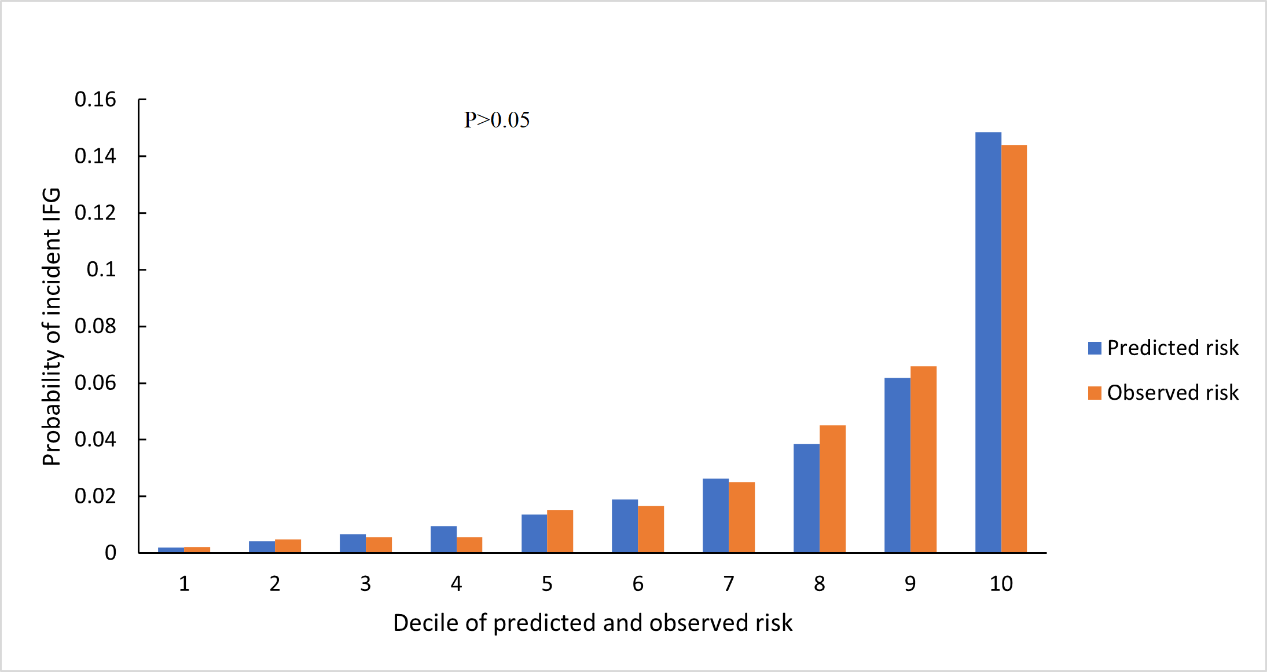


Online supplementary figure 3. Comparison between predicted and observed 5-year incidence of IFG of predicted IFG risk score for the training set in the nomogram.

A


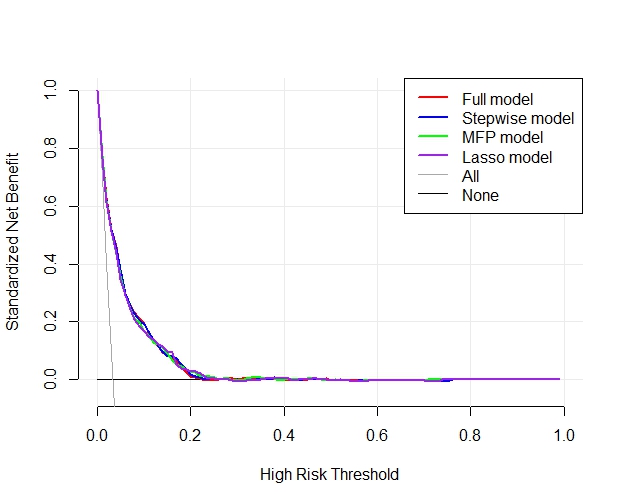


B


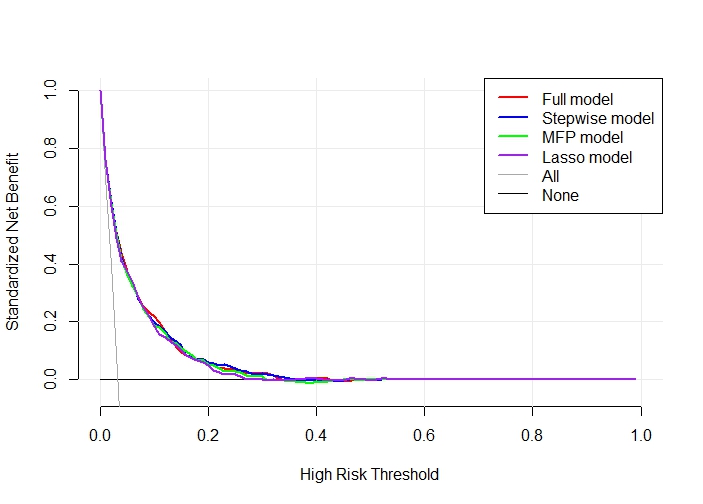


Online supplementary figure 4. The decision curve analysis of the full model, stepwise model, MFP model and LASSO model for 5-year IFG risk in the training cohort (a) and validation cohort (b). The black line represents the net benefit when none of the participants are considered to develop IFG, while the light gray line represents the net benefit when all participants are considered to develop IFG. The area between the "no treatment line" (black line) and "all treatment line" (light gray line) in the model curve indicates the clinical utility of the model. The farther the model curve is from the black and light gray lines, the better the clinical use of the nomogram.

**Online supplementary figure 5.** ROC curves with confidence intervals for LASSO, Full, Stepwise, and MFP models (Training Set)


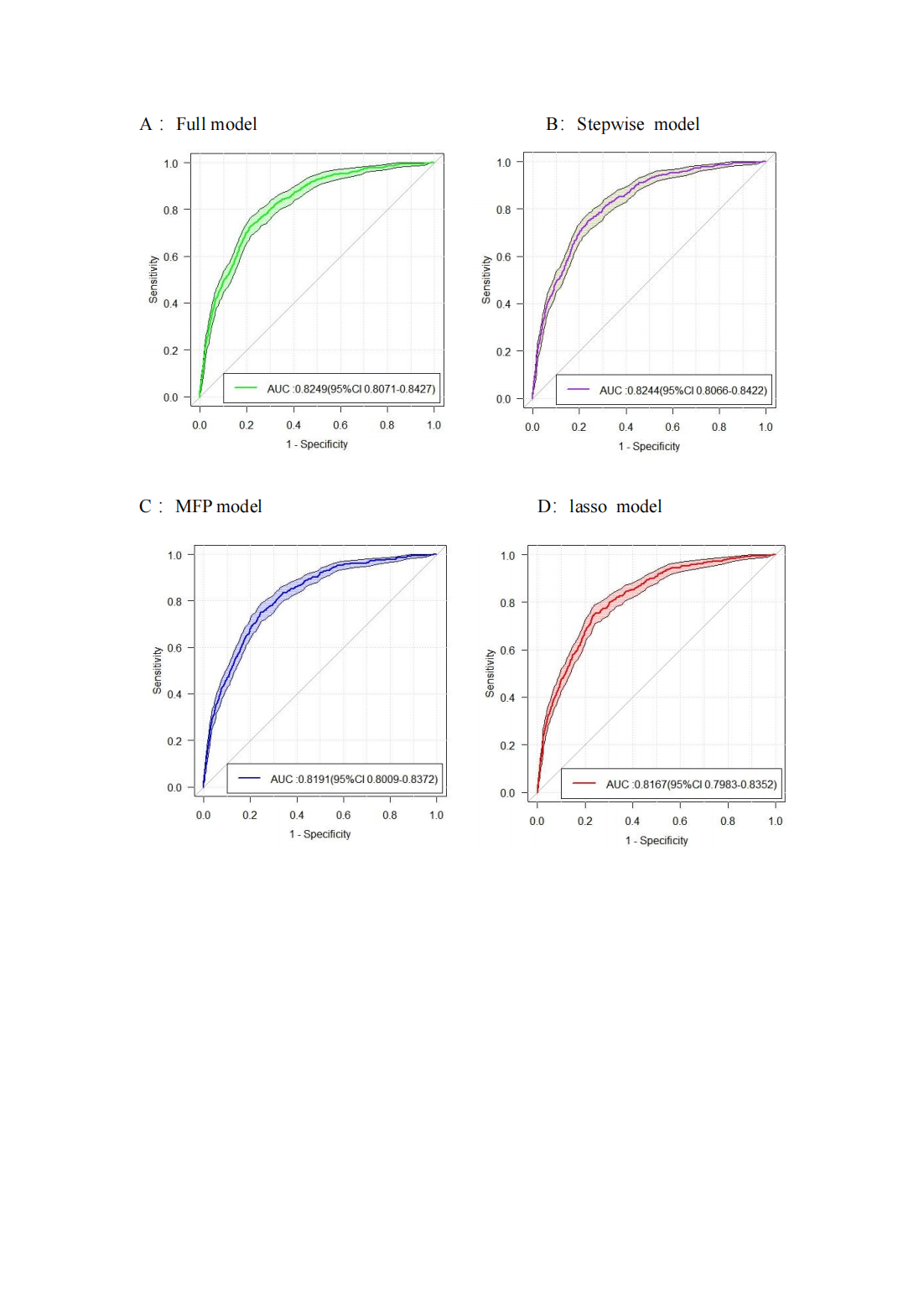


**Online supplementary figure 6.** ROC curves with confidence intervals for LASSO, Full, Stepwise, and MFP models (Validation Set)


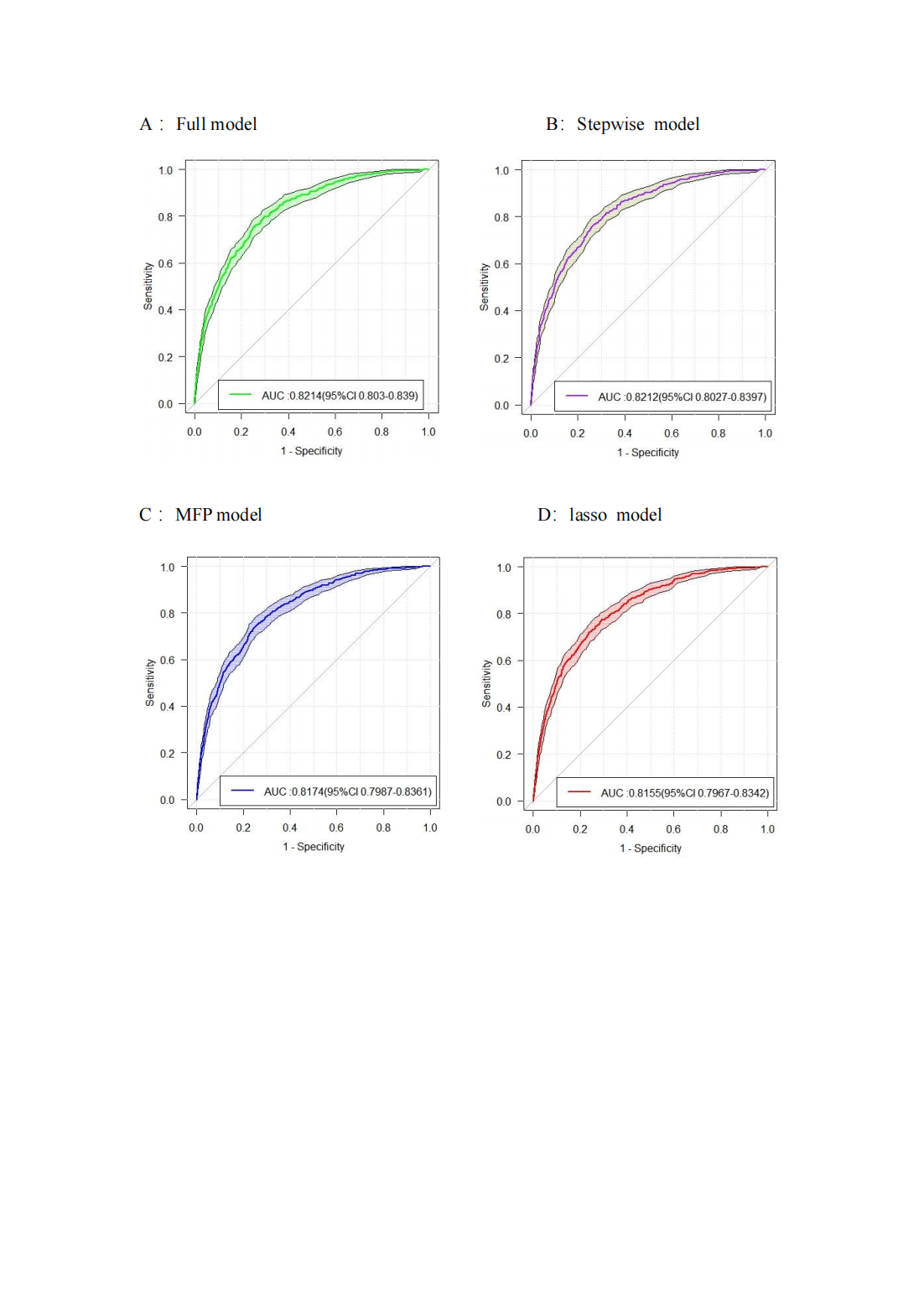


**Online supplementary figure 7.** Decision Curve Analysis with Confidence Intervals for LASSO, Full, Stepwise, and MFP models (Training Set)


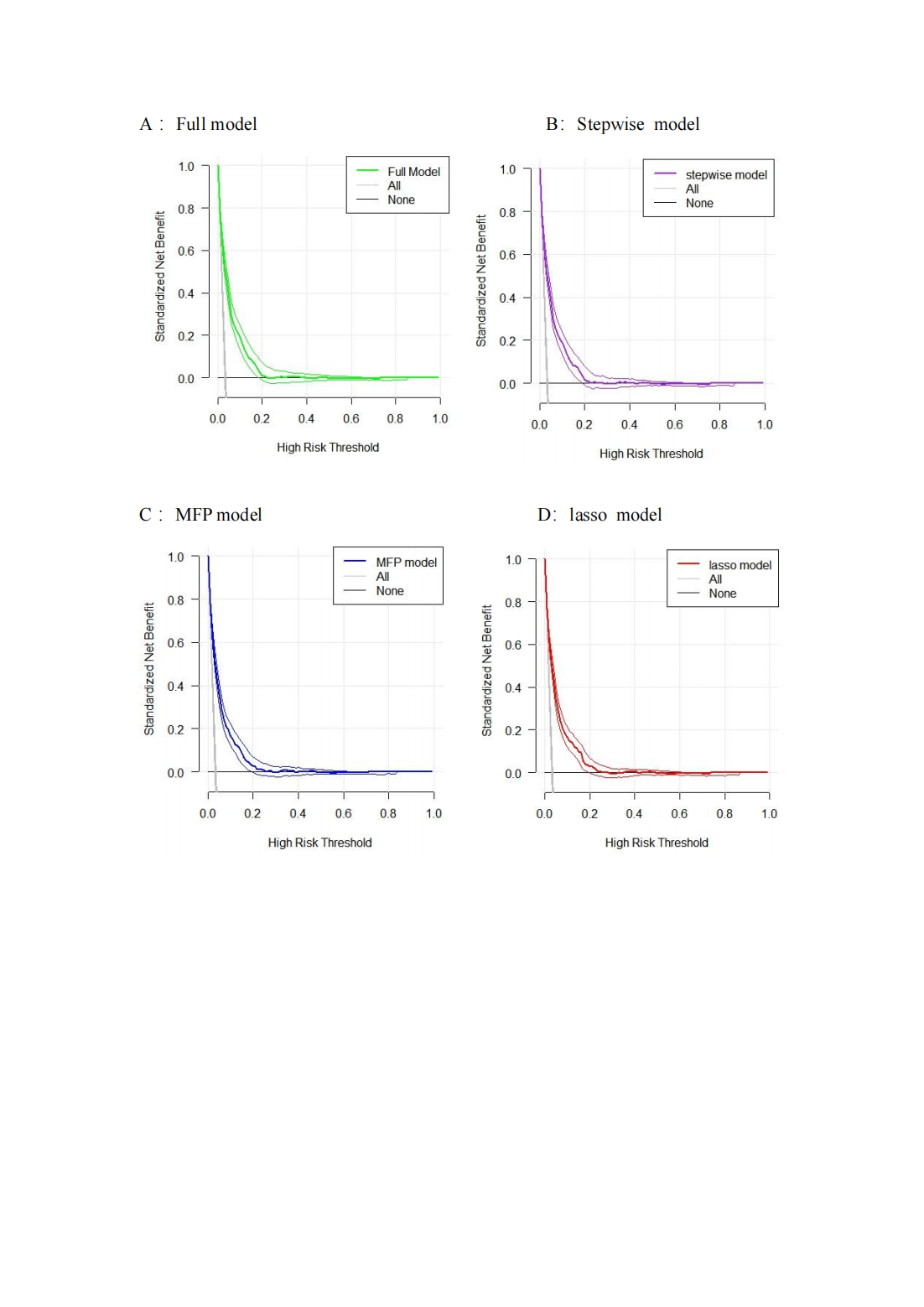


**Online supplementary figure 8.** Decision Curve Analysis with Confidence Intervals for LASSO, Full, Stepwise, and MFP models (Validation Set)


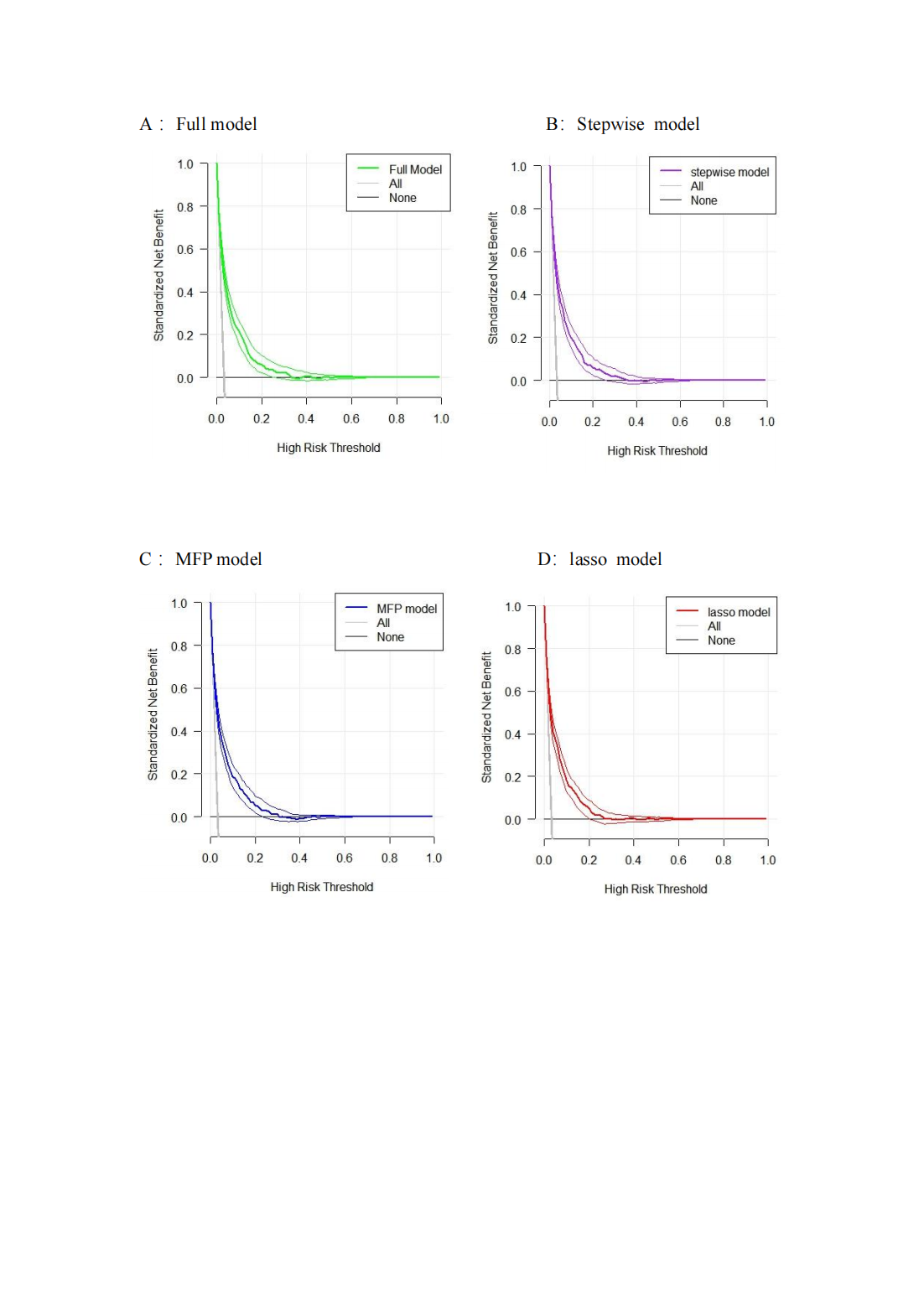

Supplement: Supplementary file 1 [file Data_Sheet_1.docx]
